# Histone deacetylase 7 mediates endothelin-1-induced connective tissue growth factor expression in human lung fibroblasts through p300 and activator protein-1 activation

**DOI:** 10.1186/s12929-021-00735-5

**Published:** 2021-05-19

**Authors:** Hung-Sheng Hua, Heng-Ching Wen, Chih-Ming Weng, Hong-Sheng Lee, Bing-Chang Chen, Chien-Huang Lin

**Affiliations:** 1 Graduate Institute of Medical Sciences, School of Medicine, College of Medicine, Taipei Medical University, Taipei, Taiwan; 2 School of Respiratory Therapy, School of Medicine, College of Medicine, Taipei Medical University, Taipei, Taiwan; 3 Department of Internal Medicine, School of Medicine, College of Medicine, Taipei Medical University, Taipei, Taiwan

**Keywords:** HDAC7, ET-1, CTGF, Airway fibrosis, Lung fibroblasts

## Abstract

**Background:**

Histone deacetylase (HDAC) inhibition was reported to ameliorate lung fibrosis in animal models. However, little is known about the underlying mechanism of HDAC7 in the regulation of CTGF production in lung fibroblasts.

**Methods:**

The role of HDAC7 in CTGF production caused by ET-1 stimulation in WI-38 cells (human lung fibroblast) was examined. We also evaluated the expression of HDAC7 in the lung of ovalbumin-induced airway fibrosis model. Statistical data were shown as mean ± standard error.

**Results:**

ET-1-stimulated CTGF and α-SMA expression was attenuated by small interfering (si)RNA interference of HDAC7. ET-1 promoted HDAC7 translocation from the cytosol to nucleus. ET-1-stimulated CTGF expression was reduced by the transfection of p300 siRNA. ET-1 induced an increase in p300 activity. Furthermore, the acetylation of c-Jun was time-dependently induced by ET-1 stimulation, which was reduced by transfection of either HDAC7 or p300 siRNA. Both transfection of HDAC7 and p300 siRNA suppressed the ET-1-increased activity of AP-1-luciferase. Moreover, the presence of HDAC7 was required for ET-1-stimulated formation of HDAC7, p300, and AP-1 complex and recruitment to the CTGF promoter region. In an ovalbumin-induced airway fibrosis model, the protein level of HDAC7 was increased in the lung tissue, and the distribution of HDAC7 was colocalized with α-SMA-positive cells in the subepithelial layer of the airway.

**Conclusions:**

ET-1 activates HDAC7 to initiate AP-1 transcriptional activity by recruiting p300 and eventually promotes the production of CTGF. HDAC7 might play a vital role in airway fibrosis and have the potential to be developed as a therapeutic target.

## Background

Asthma is defined as inflammation, airway remodeling, and airflow obstruction [5]. Patients with asthma exhibit clinical pathophysiologic characteristics including airway remodeling, subepithelial fibrosis, and epithelial detachment [1]. Subepithelial fibrosis is caused by excessive deposition of connective tissue components and extracellular matrix (ECM) in the airway wall, which in turn results in lung function impairment [34]. ECM is synthesized by fibroblasts through the expression of mediators such as connective tissue growth factor (CTGF) [41]. CTGF, formerly known as CCN2, regulates multiple biological functions such as cell proliferation, differentiation, adhesion, and matrix production as well as tissue modeling [19]. CTGF was reported to promote ECM deposition and fibroblast differentiation, and recent in vivo evidence suggested that systemic tissue fibrosis was exacerbated by CTGF overexpression [33].

The increase of ET-1 expression by the bronchial epithelium correlates with airflow obstruction and airway remodeling and characterizes severe refractory asthma [30]. In the context of tissue repair, diverse profibrotic mediators, including ET-1, recruit fibroblasts and mediate their differentiation into myofibroblasts [32]. ET-1 overexpression in the lung of mice was reported to promote myofibroblast differentiation, and the activation of focal adhesion kinase (FAK) led to ECM deposition [18]. In our previous study, we revealed that the ET_A_ receptor (ET_A_R)-dependent pathway mediates CTGF expression, which leads to fibrocyte differentiation into myofibroblasts in chronic obstructive asthma (COA) [38]. Moreover, we demonstrated that ET-1 stimulated CTGF expression through c-Jun N-terminal kinase (JNK)/activator protein (AP)-1 activation in lung fibroblasts [39].

The acetylation of transcription factors mediates transcriptional activities and regulates gene expression [8]. The balance between deacetylation and acetylation is regulated by histone deacetylases (HDACs) and histone acetyltransferases (HATs). p300 is an HAT that mediates chromatin remodeling and gene transcription [10]. Studies have suggested critical roles for p300 in modulating the epithelial-to-mesenchymal transition, myofibroblast differentiation, and production of ECM [7, 21, 40]. However, the role of p300 in CTGF expression caused by ET-1 stimulation is still unclear.

HDACs participate in fibrosis of various organs [27]. Studies have revealed that HDAC inhibitors have the potential to be used in the treatment of lung fibrosis. Trichostatin A (TSA), for example, recovered the expression of surfactant protein-C in type II alveolar epithelial cells and attenuated lung fibrosis in bleomycin-treated mice [26]. Suberoylanilide hydroxamic acid (SAHA) caused apoptosis in primary myofibroblasts of patients with idiopathic pulmonary fibrosis (IPF); moreover, SAHA reduced lung fibrosis in bleomycin-treated mice [31]. Panobinostat resulted in cell cycle arrest and apoptosis in lung fibroblasts from patients with IPF [17]. In addition to HDAC inhibitors, silencing of HDAC7 attenuated fibrotic protein expression in several studies. Silencing of HDAC7 was reported to attenuate cytokine-induced collagen I and collagen III production in primary fibroblasts from systemic sclerosis (SSc) patients [11]. HDAC7 silencing reduced TGF-β-induced Smad2 and Smad3 activation, myofibroblast differentiation, and ECM protein expression in primary fibroblasts from Peyronie’s plaque [13]. However, the involvement of HDAC7 in ET-1-induced CTGF production along with ovalbumin (OVA)-induced airway fibrosis remains unknown. In this study, we revealed that ET-1 induced the nuclear translocation of HDAC7 to form a transcriptional complex with p300 and AP-1 and mediated c-Jun acetylation through p300 activity, followed by the recruitment of the complex to the CTGF promoter region, which in turn promoted CTGF production. Moreover, the expression of HDAC7 in the lung of an OVA-induced airway fibrosis mouse model was elevated.

## Materials and methods

### Materials

WI-38 normal human embryonic lung fibroblast cell lines (ATCC CCL-75) were purchased from American Type Culture Collection (Manassas, VA, USA). Recombinant human ET-1 was obtained from Bachem Americas (Torrance, CA, USA). Minimum essential medium (MEM), Lipofectamine 3000 reagent, and fetal bovine serum (FBS) were bought from Invitrogen Life Technologies (Carlsbad, CA, USA). The chromatin immunoprecipitation (IP; ChIP) assay kit was obtained from Upstate Biotechnology (Lake Placid, NY, USA). All materials for Western blotting were acquired from Bio-Rad (Hercules, CA, USA). Antibodies specific for CTGF and antigoat, antimouse, and antirabbit immunoglobulin G (IgG)-conjugated horseradish peroxidase (HRP) were procured from Santa Cruz Biotechnology (Santa Cruz, CA, USA). Anti-α-tubulin antibody was bought from MilliporeSigma (Burlington, MA, USA). HDAC7 and α-SMA antibodies were purchased from Abcam (Cambridge, MA, USA). Antibodies specific for c-Jun, acetyl-lysine, and p300 were bought from Cell Signaling Technology (Danvers, MA, USA). Lamin A/C antibody was procured from GeneTex (Irvine, CA, USA). pAP-1-Luc *Cis*-reporter plasmid was obtained from Stratagene (Santa Clara, CA, USA). pBK-CMV-*Lac Z* (*LacZ*) was obtained from Dr. W-W. Lin (National Taiwan University, Taipei, Taiwan). The luciferase assay system was procured from Promega (Madison, WI, USA). The cytoplasmic and nuclear protein extraction kit was bought from BIOTOOLS (BIOTOOLS, New Taipei City, Taiwan). OVA was bought from MilliporeSigma. Aluminum hydroxide was bought from Thermo Fisher Scientific (Waltham, MA, USA). All other chemicals were bought from MilliporeSigma.

### Cell culture

WI-38 cells were cultured in MEM supplemented with 10% FBS in a CO_2_ incubator at 37 °C. After reaching confluence, cells were seeded into 6-cm dishes for cell transfection and immunoblotting, 10-cm dishes for the ChIP assay, and 12-well plates for the luciferase assay and cell transfection. For experiments, cells at passages 20–32 were used.

### Transfection and luciferase reporter assays

WI-38 cells (5 × 10^4^ cells/well) were cultured in 12-well plates overnight. The cells were transfected with 0.5 μg of AP-1-Luc, CTGF-Luc, and *Lac Z* with Lipofectamine 3000. After 6 h, the medium was replaced with serum-free medium overnight. The cells were incubated with ET-1 (10 nM) for 16 h and then collected to measure the luciferase activity. The expression of LacZ was used to normalize luciferase activity. Levels of increase in luciferase activity were compared as the ratio of cells with and without stimulation.

### Western blot analysis

After ET-1 stimulation, the cells were collected. Whole-cell lysates (30 μg) were separated through sodium dodecyl sulfate polyacrylamide gel electrophoresis (SDS-PAGE) and transferred to a polyvinylidene difluoride (PVDF) membrane. The membrane was immersed in nonfat milk (5%) at room temperature for 1 h and then immersed in primary antibodies at 4 °C overnight. Proteins were detected using antibodies specific for CTGF, HDAC7, p300, c-Jun, acetyl-lysine, α-SMA, α-tubulin, or lamin A/C. Subsequently, the membrane was immersed in HRP-conjugated secondary antibody at room temperature for 1 h. The blots were manifested using ECL reagents. Scientific imaging system (Kodak, Rochester, NY, USA) was applied to obtain quantitative data.

### Immunofluorescence staining

WI-38 cells were seeded onto coverslips in 6-cm culture dishes. The cells were treated with paraformaldehyde (4%) for 20 min after ET-1 (10 nM) stimulation. This was followed by incubation with 5% bovine serum albumin at room temperature for 1 h and then incubation with DAPI and an antibody specific for HDAC7 at room temperature for 2 h. The cells were then incubated with an Alexa-488-conjugated secondary antibody for 1 h to detect immunoreactivity.

The paraffin-embedded mouse lung tissue sections were dewaxed in xylene and alcohols. α-SMA was immunostained using an antibody specific for α-SMA first and then using an Alexa-555-conjugated secondary antibody. In addition, HDAC7 was immunostained using an antibody specific for HDAC7, followed by immunostaining with an Alexa-488-conjugated secondary antibody. The DAPI dye was used as a nuclear stain.

### Coimmunoprecipitation

WI-38 cells were seeded in 10-cm dishes and treated with ET-1 (10 nM). The cells were harvested and centrifuged at 15,000 × *g* at 4 °C for 10 min. The supernatants were immunoprecipitated with antibodies specific for HDAC7, c-Jun, or p300 through the use of protein A beads overnight at 4 °C. Samples were then analyzed using Western blotting.

### ChIP assay

WI-38 cells were seeded in 10 cm dishes and incubated with ET-1 (10 nM) for 20 min. After ET-1 stimulation, cells were incubated with 1% formaldehyde at room temperature for 10 min and then collected. The samples were sonicated and then centrifuged at 15,000 × *g* at 4 °C for 10 min. The supernatants were immunoprecipitated with antibodies specific for rabbit IgG, c-Jun, HDAC7, or p300 in the presence of protein A beads at 4 °C overnight. DNA was purified with a spinning filter and then was eluted with 50 μL of double-distilled (dd)H_2_O. The AP-1 response element on the promoter region of CTGF was amplified using a polymerase chain reaction (PCR). The sequences of primers were as follows: 5′-CGT CCC TTG TCC TTG CCT AT-3′ (sense) and 5′-GCT CGA CCT CAC ACG GTC GA-3′ (antisense). The PCR conditions were as follows: 40 cycles of amplification at 95 °C for 30 s, 62 °C for 60 s, and 72 °C for 30 s. Agarose gel (2%) electrophoresis was used to analyze the PCR products.

### Sensitization and antigen challenge protocol

Female C57BL/6 mice (BioLASCO, Taipei, Taiwan), aged 7 weeks old, were used in the experiments. They were divided into two groups (n = 6 per group): control (phosphate buffered saline, PBS) and OVA treatment. The mice were immunized by subcutaneous injection on days 1, 8, and 15 with 50 μg of OVA adsorbed to 4 mg of aluminum hydroxide in 200 μL of PBS. OVA challenges (20 μg/50 μL in PBS) were started on day 28 and were repeated twice a week for 8 weeks in an ultrasonic nebulizer chamber. The control mice were treated in the same way with PBS but without OVA. The mice were sacrificed at 12 weeks. All animal experimental protocols were approved by Taipei Medical University Institutional Animal Care and Use Committee (LAC-101-0243).

### Statistical analysis

At least three independent experiments were conducted. Data are presented as mean ± standard error of the mean (SEM). A one-way ANOVA was conducted, followed by Dunnett’s test. In all cases, *p* < 0.05 was considered statistically significant.

## Results

### HDAC7 regulated ET-1-stimulated CTGF and α-SMA expression in WI-38 cells

**Fig. 1:**
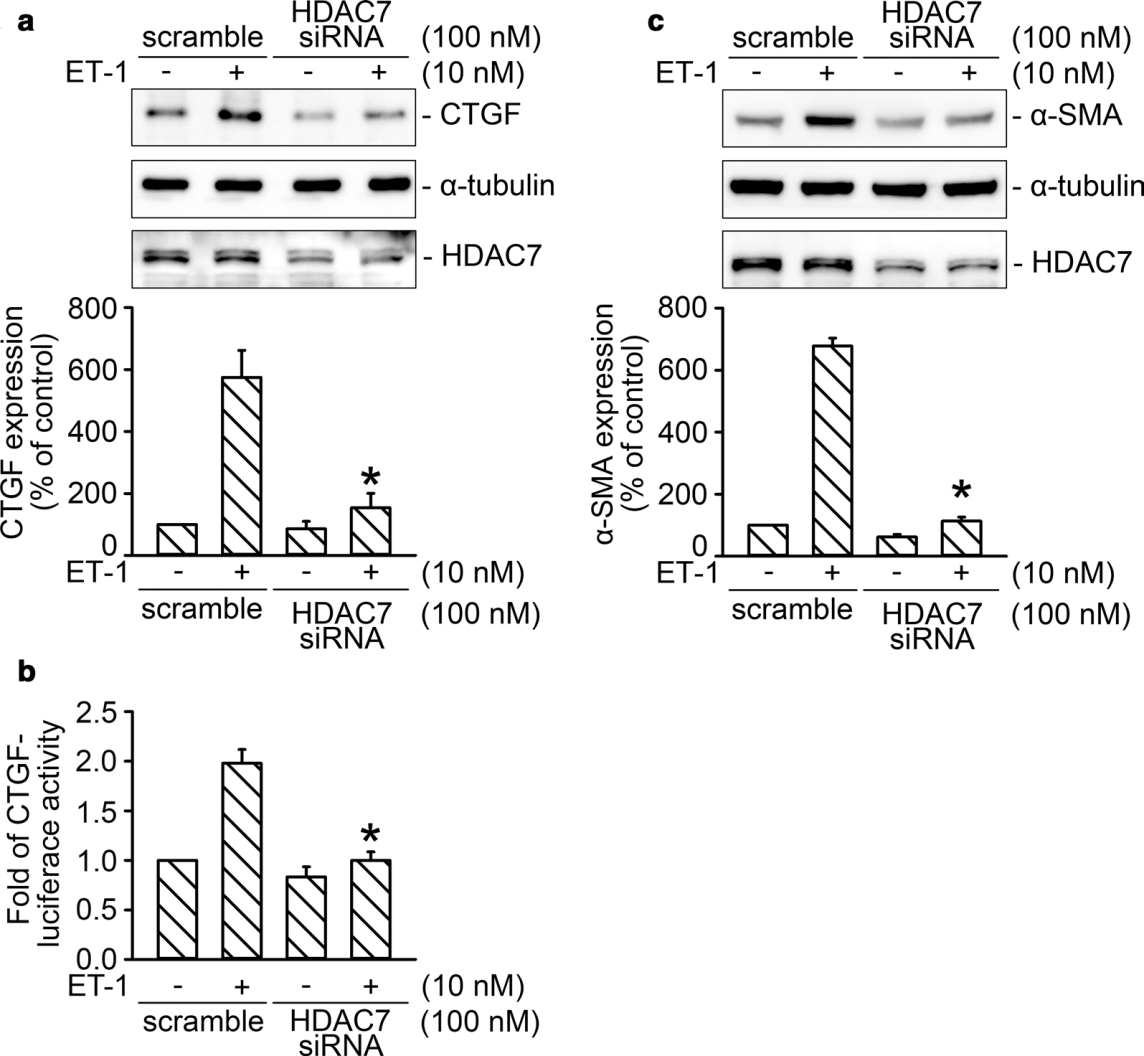
HDAC7 participates in CTGF and α-SMA expression induced by ET-1 stimulation in WI-38 cells. **A** HDAC7 was knocked down by transfection with small interfering (si)RNA for 24 h. After 2 h of treatment by ET-1, the protein level of CTGF in the WI-38 cells was evaluated using Western blot analysis. α-Tubulin acted as loading control. Bars represent values of the mean ± SEM (*n* = 5). *p < 0.05, compared with the ET-1 treatment group. **B** Cells were transfected with CTGF-Luc (1 μg), Lac Z (1 μg), and HDAC7 siRNA for 24 h and then stimulated by ET-1 treatment for 16 h. Luciferase activity was evaluated as described in “Materials and methods.” Bars represent values of the mean ± SEM (*n* = 4). *p < 0.05, compared with ET-1 treatment. **C**, After transfection of HDAC7 siRNA for 24 h, cells were treated with ET-1 for 48 h, and then α-SMA expression was analyzed using immunoblotting. α-Tubulin was used as loading control. Bars represent values of the mean ± SEM (*n* = 5)

HDAC7 mediates collagen production and is involved in the fibrosis of SSc [11]. α-SMA is the hallmark of myofibroblasts, and its levels are significantly increased in lung fibrosis [23]. In this study, siRNA technology was used to assess the role of HDAC7 in the expression of both CTGF and α-SMA, induced by ET-1 stimulation in WI-38 cells. siRNA interference of HDAC7 markedly reduced ET-1-induced CTGF production by 89% ± 9% (*n* = 5; Fig. 1A). HDAC7 siRNA (100 nM) also suppressed ET-1-induced activity of CTGF-luciferase by 103% ± 10% (*n* = 4; Fig. 1B). In addition, HDAC7 siRNA (100 nM) suppressed α-SMA expression induced by ET-1 stimulation by 98% ± 2% (*n* = 5; Fig. 1C). The results indicate that HDAC7 positively regulated ET-1-stimulated expression of both CTGF and α-SMA.

### ET-1 stimulated HDAC7 activation in WI-38 cells

**Fig. 2:**
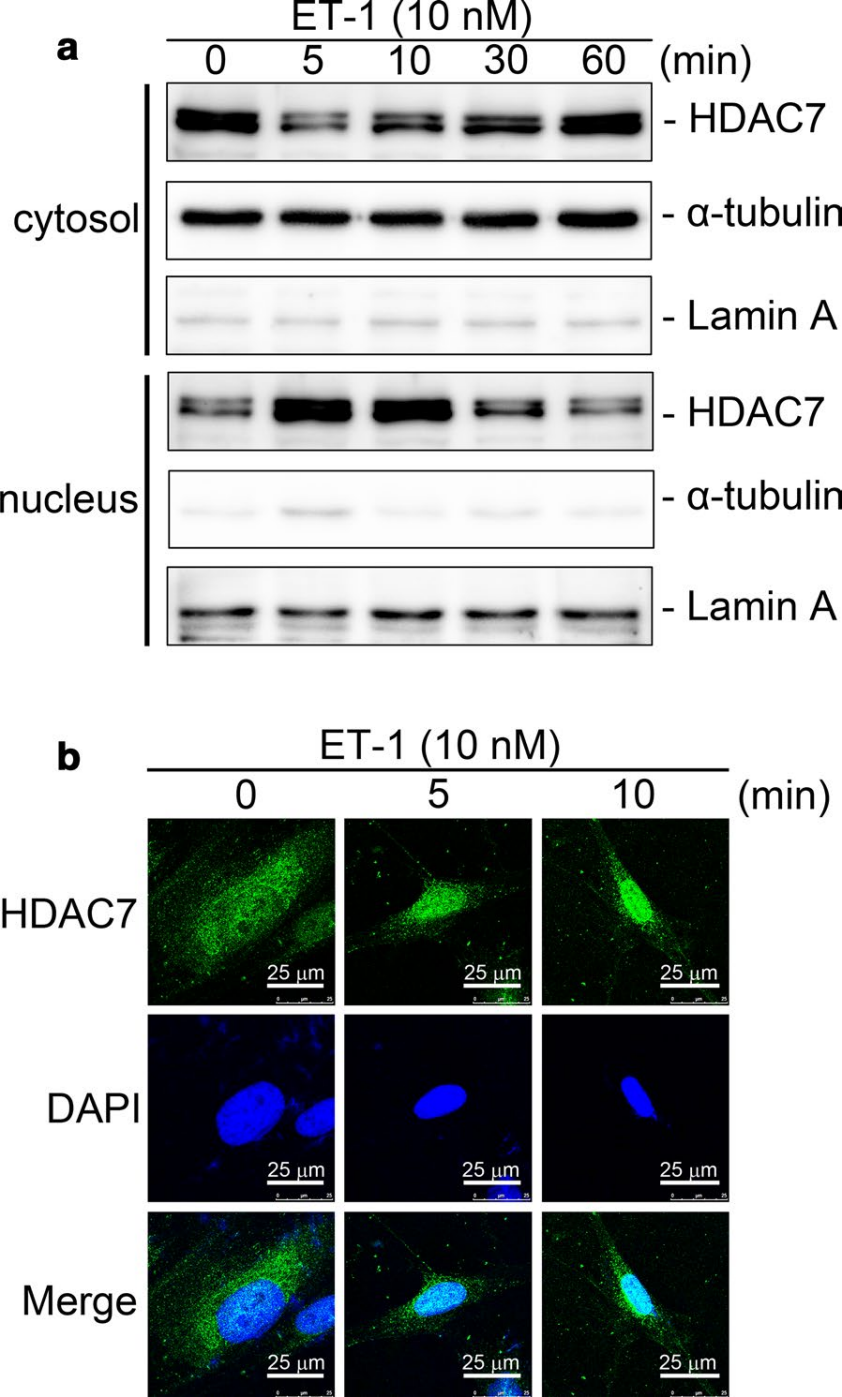
ET-1 induces the nuclear translocation of HDAC7 in WI-38 cells.** A** After ET-1 stimulation for 0, 5, 10, 30, and 60 min, cells were collected. The distribution of HDAC7 was analyzed using a cytoplasmic and nuclear protein extraction kit and Western blotting (*n* = 4). Lamin A and α-tubulin were loading controls. **B** After ET-1 stimulation for 0, 5, and 10 min, cells were immunodetected with an antibody specific for HDAC7 (green); nuclei were detected with DAPI (blue) (*n* = 3). IF staining is described in “Materials and methods.”

Signal-dependent subcellular localization plays a vital role in regulating the activities of HDAC7 [14]. To evaluate whether ET-1 promotes HDAC7 nuclear localization, nuclear extraction and immunofluorescence (IF) staining were used. Incubation of cells with ET-1 (10 nM) for 5–60 min induced HDAC7 translocation from the cytosol to nuclei, with a maximum effect at 5 and 10 min after ET-1 stimulation (Fig. 2A). Similarly, IF staining revealed that nuclear translocation of HDAC7 increased at 5 and 10 min after ET-1 stimulation (Fig. 2B). These results suggest that HDAC7 was activated by ET-1 treatment.

### Involvement of p300 in CTGF production caused by ET-1 stimulation

**Fig. 3:**
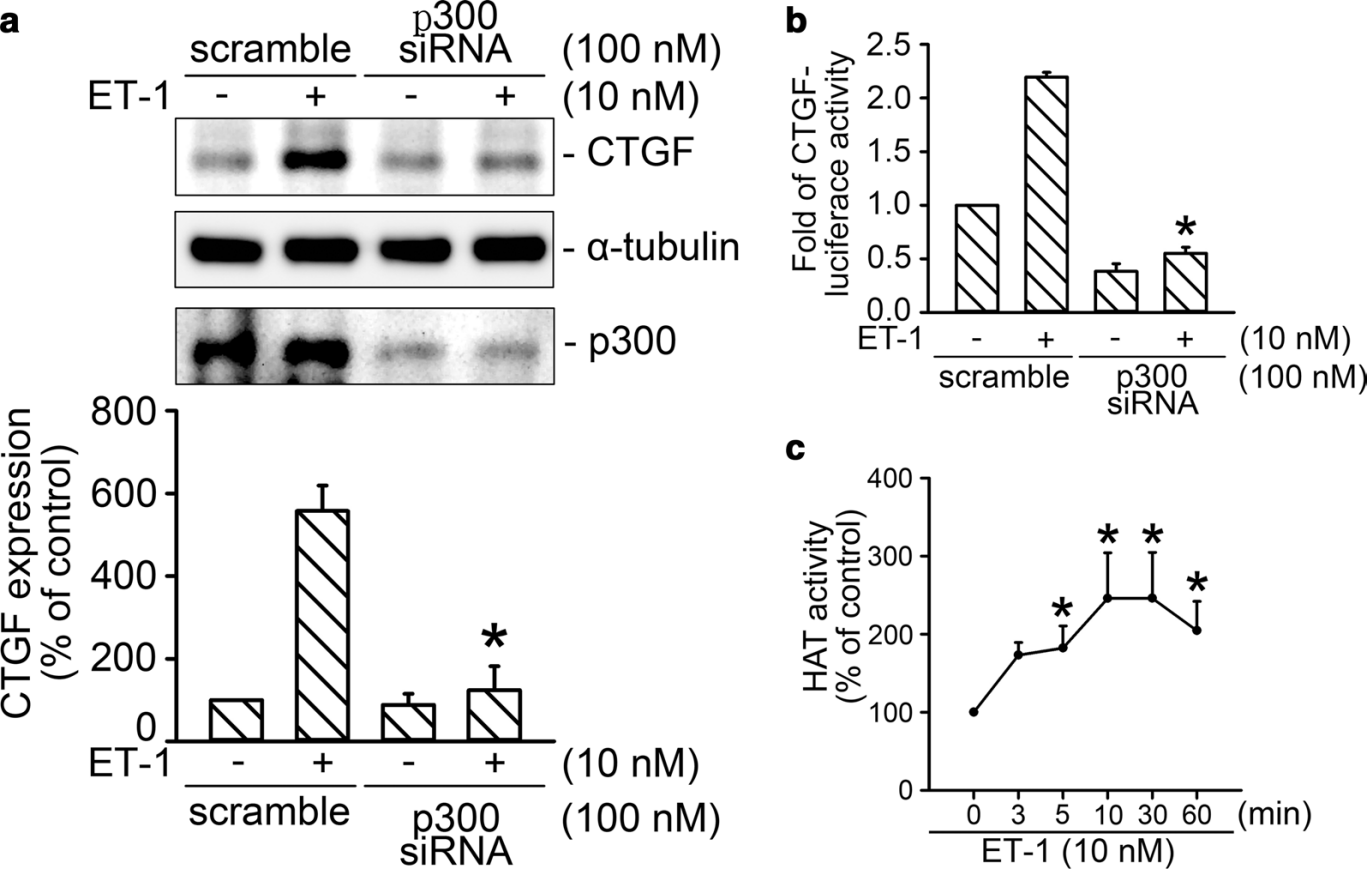
p300 participates in the expression of CTGF caused by ET-1 stimulation in WI-38 cells.** A** p300 was knocked down by transfection of siRNA for 24 h. ET-1 stimulation proceeded for 2 h. Western blot analysis was conducted to detect the protein level of CTGF. α-Tubulin was the loading control. Bars represent values of the mean ± SEM (*n* = 4). *p < 0.05, compared with the ET-1 stimulation group. **B** After transfection with CTGF-Luc (1 μg), Lac Z (1 μg), and p300 siRNA for 24 h, the cells were stimulated by ET-1 for 16 h. The activity of luciferase was measured as described above. Bars represent values of the mean ± SEM (*n* = *6*). *p < 0.05, compared with ET-1 treatment group. **C**, After ET-1 stimulation for various time intervals, histone acetyltransferase activity was analyzed. Data are presented as mean ± SEM (*n* = 3). *p < 0.05, compared with the control without ET-1 stimulation as 100%

p300 was reported to participate in TGF-β-induced CTGF production in mouse embryonic fibroblasts [42]. To evaluate whether p300 participates in CTGF production caused by ET-1 treatment, p300 siRNA was used. siRNA interference of p300 reduced ET-1-induced CTGF protein level by 99 ± 14% (*n* = 4; Fig. 3A). ET-1-stimulated activity of CTGF-luciferase was also suppressed by p300 siRNA (100 nM) by 138 ± 4% (*n* = 6; Fig. 3B). We further determined that ET-1 (10 nM) treatment time-dependently increased the activity of HAT (Fig. 3C). Induction of HAT activity had begun by 3 min and reached a maximum at 10–30 min after ET-1 treatment (Fig. 3C). These results suggest that activation of p300 contributed to ET-1-stimulated CTGF expression.

### HDAC7 and p300 participated in ET-1-induced activation of AP-1

**Fig. 4:**
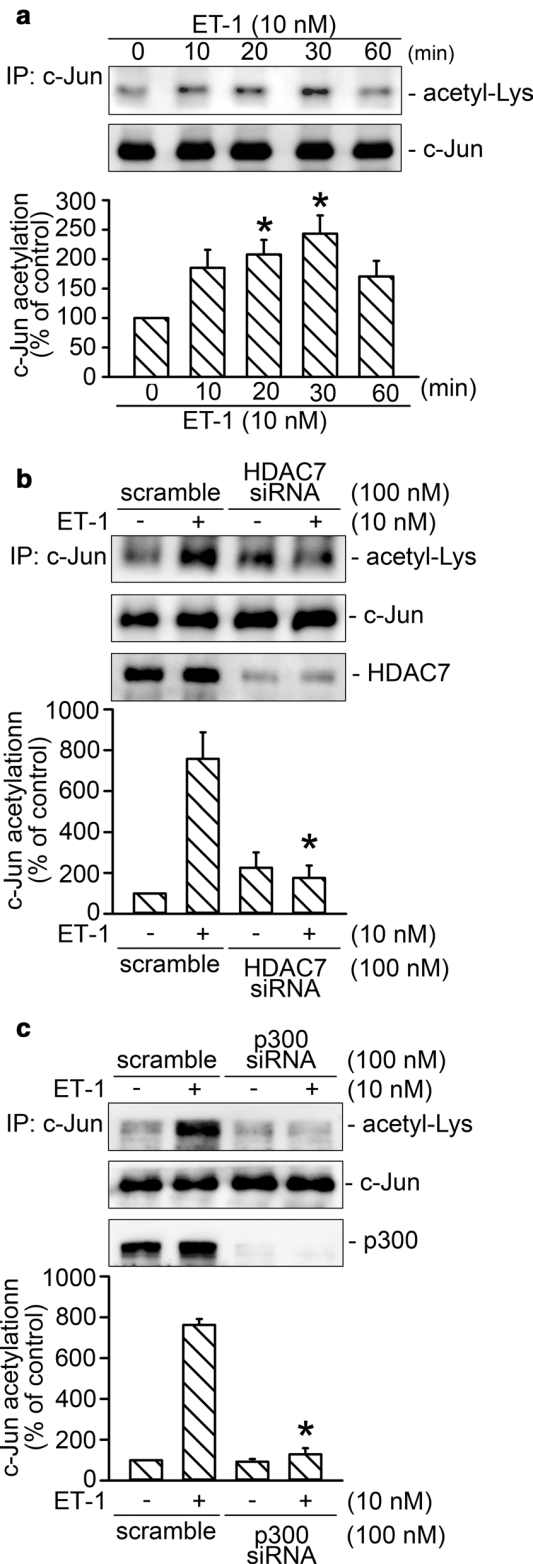
HDAC7 participated in ET-1-stimulated c-Jun acetylation in WI-38 cells.** A** Cells were stimulated by ET-1 treatment for 0, 10, 20, 30, and 60 min. After ET-1 treatment, immunoprecipitation was performed with c-Jun antibody. Western blotting was conducted to detect the acetylation of c-Jun. Bars represent values of the mean ± SEM (*n* = 5). *p < 0.05, compared with the control without ET-1 treatment. After transfection with HDAC7 (B) or p300 siRNA (C) for 24 h, cells were stimulated by ET-1 treatment for 20 min. Immunoprecipitation and Western blotting were conducted to detect the acetylation of c-Jun. Bars represent values of the mean ± SEM (*n* = 4) *p < 0.05, compared with the ET-1 treatment group

**Fig. 5:**
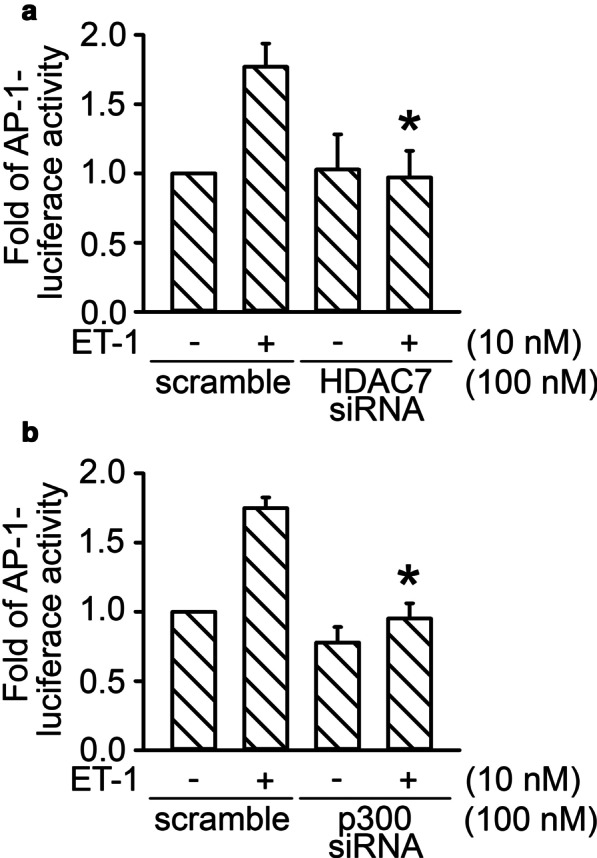
Involvement of HDAC7 and p300 in ET-1-stimulated AP-1 activation in WI-38 cells. **A** After transfection with AP-1-Luc (1 μg), Lac Z (1 μg), and HDAC7 siRNA for 24 h, cells were treated with ET-1 for 16 h. Luciferase activity was evaluated as described in “Materials and methods.” Bars represent values of the mean ± SEM (*n* = 3). *p < 0.05, compared with ET-1 treatment. **B** After transfection with AP-1-Luc (1 μg), Lac Z (1 μg), and p300 siRNA for 24 h, the cells were treated with ET-1 for 16 h. Luciferase activity was measured as described in “Materials and methods.” Bars represent values of the mean ± SEM (*n* = 3). *p < 0.05, compared with ET-1 treatment

HDAC7 mediates the activation of HIF-1α through a complex formation with HDAC7 and p300 [15]. Moreover, p300 is involved in the EGF-stimulated expression of *keratin 16* through the regulation of c-Jun acetylation in HaCaT cells [37]. In this study, the involvement of HDAC7 in ET-1-stimulated c-Jun acetylation was investigated. After ET-1 (10 nM) stimulation, the acetylation of c-Jun time-dependently increased and achieved a maximum at 30 min (*n* = 5; Fig. 4A). Transfection of HDAC7 siRNA (100 nM) reduced ET-1-induced c-Jun acetylation by 90% ± 6% (*n* = 4; Fig. 4B). Transfection of p300 siRNA (100 nM) reduced ET-1-induced c-Jun acetylation by 96% ± 4% (n = 4; Fig. 4C).The results suggest that HDAC7 and p300 mediated ET-1-induced c-Jun acetylation. To further ascertain the roles of HDAC7 and p300 in mediating AP-1 activity, we determined the effects of HDAC7 and p300 siRNA on ET-1-induced activity of AP-1-luciferase. Transfection of HDAC7 siRNA (100 nM) and p300 siRNA (100 nM) suppressed ET-1-stimulated AP-1-luciferase activity by 113 ± 25% (*n* = 3) and 110 ± 17% (*n* = 3), respectively (Fig. 5A and B). Taken together, the results reveal that HDAC7 and p300 regulated AP-1 activation caused by ET-1 stimulation in lung fibroblasts.

### ET-1 stimulated the formation of HDAC7, p300, and AP-1 complex and was then recruited to the AP-1 binding site on CTGF promoter

**Fig. 6:**
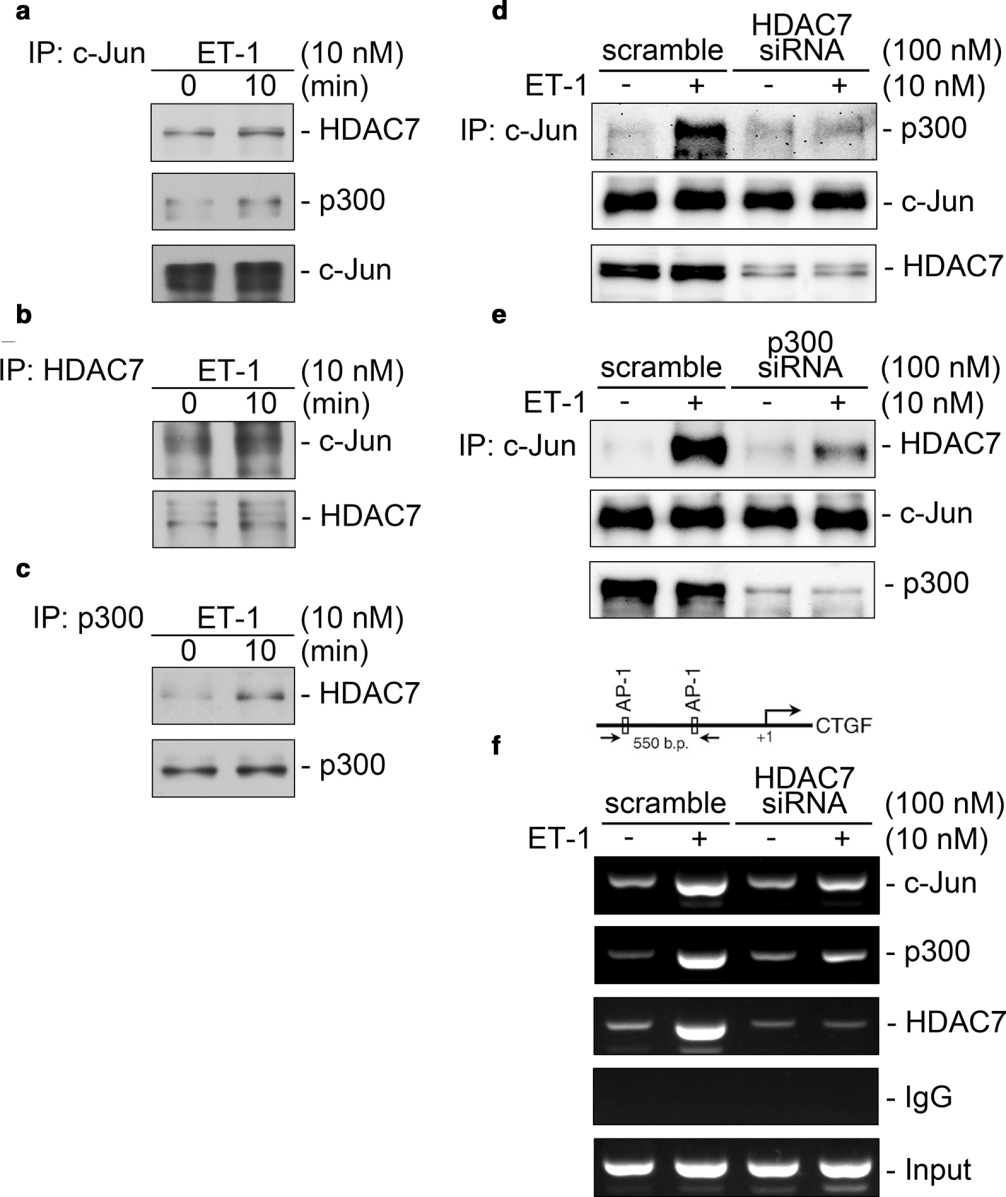
ET-1 promotes the complex formation of HDAC7, p300, and AP-1 in WI-38 cells. After ET-1 (10 nM) stimulation for 10 min, cells were collected and then immunoprecipitated with antibodies specific for c-Jun (**A**), HDAC7 (**B**), or p300 (**C**). Samples were analyzed using Western blotting. Equal loading in each lane is shown by the similar intensities of c-Jun, HDAC7, or p300 (*n* = 3). **D** After transfection with HDAC7 siRNA for 24 h, cells were stimulated by ET-1 treatment for 10 min. Immunoprecipitation and Western blotting were conducted to detect the protein–protein interaction between c-Jun and p300 (n = 4). E, After transfection with p300 siRNA for 24 h, cells were stimulated by ET-1 treatment for 10 min. Immunoprecipitation and Western blotting were conducted to detect the protein–protein interaction between c-Jun and HDAC7 (n = 4). F Schematic of the 550-bp ChIP primer located on the CTGF promoter. After transfection with HDAC7 siRNA for 24 h, cells were stimulated by ET-1 treatment for 20 min, and ChIP assay was conducted as described in “Materials and methods.” Equal amounts of each PCR were confirmed by the product for input. Nonimmune IgG was used as a negative control (n = 4)

To evaluate whether HDAC7 regulates CTGF expression through connection with p300 and AP-1, Co-IP and ChIP assays were conducted on ET-1-stimulated WI-38 cells. The Co-IP assay revealed that ET-1 induced an increase in protein–protein interaction among HDAC7, p300, and c-Jun (Fig. 6A–C). Transfection of HDAC7 siRNA attenuated ET-1-induced protein–protein interaction between c-Jun and p300 (Fig. 6D). In addition, transfection of p300 siRNA suppressed ET-1-induced HDAC7/c-Jun interaction (Fig. 6E). To determine whether the HDAC7, p300, and AP-1 complex is recruited to the promoter region of CTGF, we conducted ChIP assays. The recruitment of HDAC7, p300, and c-Jun to the AP-1 binding site on the CTGF promoter region increased after ET-1 stimulation. Furthermore, transfection of HDAC7 siRNA attenuated the binding of c-Jun, p300, and HDAC7 to CTGF promoter (Fig. 6F). These results reveal that ET-1 induced the formation of the HDAC7, p300, and AP-1 complex, followed by the complex binding to the AP-1 response element on the CTGF promoter. Moreover, HDAC7 is required for the complex formation of HDAC7/p300/c-Jun and the recruitment of the complex to CTGF promoter upon ET-1 stimulation.

### HDAC7 expression was increased and colocalized with α-SMA-positive cells in OVA-induced airway fibrosis

**Fig. 7:**
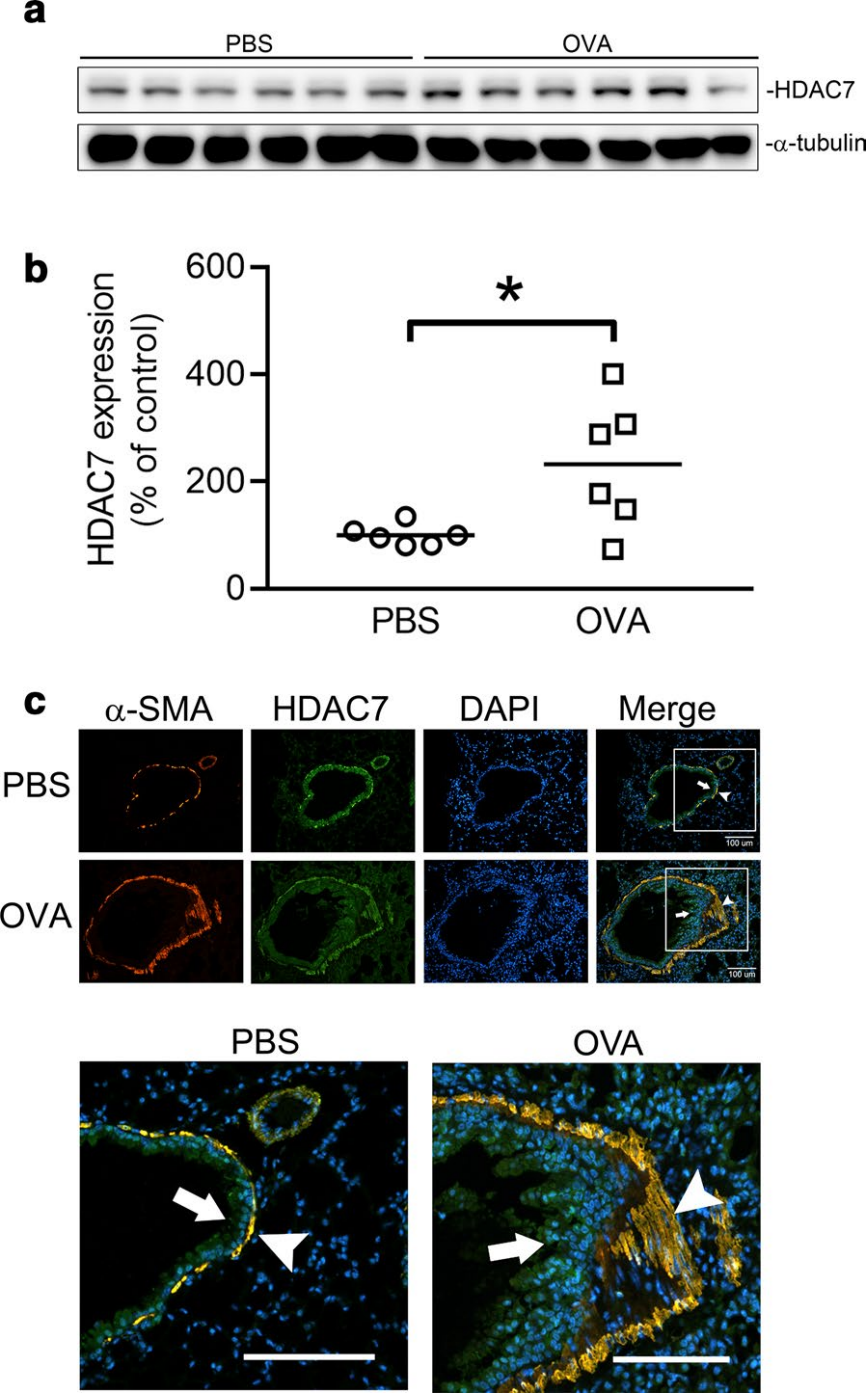
HDAC7 expression is elevated in the lung of ovalbumin (OVA)-induced airway fibrosis in mice. **A** Lung from PBS- or OVA-treated mice was homogenized, and the expression of HDAC7 was analyzed through Western blotting. α-Tubulin was the loading control. **B** Statistical analysis of the results of Western blotting. Each dot represents an individual mouse, and horizontal lines indicate mean values (*n* = 6). *p < 0.05, compared with the PBS group. **C** Paraffin sections of lung tissue from the PBS- or OVA-treated mice were stained for α-SMA (red), HDAC7 (green), and nuclei (blue). The red area represents α-SMA-positive cells, the green area represents HDAC7-positive cells, and the yellow area represents the colocation of α-SMA and HDAC7. The epithelial cell layer is labeled by a white arrow. The subepithelial layer is labeled by a white arrowhead. The IF staining and microscope analysis are described in “Materials and methods.” Bar, 100 μm

We established a mouse model of OVA-induced airway fibrosis to evaluate the role of HDAC7 in the pathogenesis of airway fibrosis. The protein level of HDAC7 was analyzed using Western blotting. Distributions of HDAC7 and α-SMA were evaluated through IF staining. The expression of HDAC7 was elevated in the lung tissue from OVA-treated mice compared with the control group (*n* = 6; Fig. 7A and B). The distributions of α-SMA and HDAC7 in the lung sections from the mice were examined using IF staining. The α-SMA was expressed in the subepithelial layer, and the HDAC7 was expressed in both epithelial cells and the subepithelial layer. In the OVA-treated mice, HDAC7 was markedly elevated in the subepithelial layer compared with the control group and was colocalized with α-SMA (Fig. 7C). These results reveal that HDAC7 might play a vital role in OVA-induced airway fibrosis in mice.

## Discussion

ET-1 plays a pathological role in lung diseases [9], and several pieces of evidence have indicated that ET-1 also acts as a profibrotic mediator [2, 25]. The results of our previous study suggested that ET-1 induces CTGF and α-SMA expression through the ET_A_R/JNK/AP-1 signaling pathway, and CTGF was required for ET-1-stimulated myofibroblast differentiation [39]. In this study, ET-1 stimulated the nuclear translocation of HDAC7 and increased the HAT activity of p300, which in turn promoted c-Jun acetylation and AP-1 activation, and eventually promoted CTGF production. In OVA-induced airway fibrosis, the protein level of HDAC7 was elevated in the subepithelial layer of the airway, and HDAC7 was colocalized to α-SMA-positive cells. HDAC7 might be essential to the pathogenesis of airway fibrosis.

HDAC7, which belongs to the class IIa HDAC family, has been shown to participate in several fibrotic diseases. One study indicated that increased expression of HDAC7 promoted profibrotic gene expression during the activation of hepatic stellate cells [6]. HDAC7 was also involved in cytokine-induced collagen production in fibroblasts from SSc patients [11]. The expression of HDAC7 was increased in the lung of IPF patients [16]. HDAC7 was reported to be involved in TGF-β-mediated activation of fibroblasts in patients with IPF [12]. Class IIa HDAC activities are regulated by nuclear/cytoplasm shuttling [4]. A previous study indicated that myosin phosphatase promotes HDAC7 nuclear localization by mediating its dephosphorylation, leading to the suppression of apoptosis in CD4^+^CD8^+^ thymocytes [28]. In addition, HDAC7 translocates into the nucleus and enhances HIF-1α transcription activity under hypoxia in HEK293 cells [15]. In the present study, the transfection of HDAC7 siRNA attenuated the ET-1-stimulated protein level of CTGF, activity of CTGF-luciferase, and protein level of α-SMA in human lung fibroblasts. We also found that ET-1 stimulates the translocation of HDAC7 from the cytosol to nucleus. The results suggest that HDAC7 is involved in ET-1-stimulated CTGF production and myofibroblast differentiation.

p300 is a component of the chromatin remodeling and transcriptional complex that modulates gene expression [10]. A previous study indicated that p300 is upregulated in the lung of IPF patients [35]. Moreover, p300 was involved in TGF-β-stimulated collagen and fibronectin synthesis in lung fibroblasts [35]. p300 was reported to mediate thrombin-induced fibrotic cytokine CCL2 expression through histone acetylation and NF-κB activation in lung fibroblasts [3]. In this study, ET-1-induced CTGF protein level and the activity of CTGF-luciferase were reduced by the siRNA interference of p300. Furthermore, ET-1 time-dependently stimulated HAT activity. These results suggest that p300 activation participates in ET-1-induced CTGF production.

The enzymatic activity of HAT enables p300 to regulate the acetylation of histone and to promote gene expression [36]. p300 can also regulate the acetylation of transcription factors. A previous study established that p300 is a cofactor for AP-1 subunit c-Jun [20]. EGF promoted *keratin 16* expression through p300-mediated acetylation of c-Jun in HaCaT cells [37]. HDAC7 associated with HIF-1α to induce the acetylation of HIF-1α through the HAT activity of p300 under hypoxia stimulation. [15]. In this study, we found that ET-1 stimulated c-Jun acetylation in a time-dependent manner. Transfection of either HDAC7 or p300 siRNA attenuated ET-1-stimulated c-Jun acetylation. Furthermore, transfection of either HDAC7 or p300 siRNA suppressed the ET-1-induced activity of AP-1-luciferase. HDAC7 was reported to act as a transcriptional coactivator through the regulation of HIF-1α activity [15]. Moreover, p300 mediated c-Jun transcriptional activity through regulation of c-Jun acetylation in glioma cells [22]. In the present study, we speculate that HDAC7 and p300 might play a role as a coactivator in ET-1-stimulated AP-1 activation through the modulation of c-Jun acetylation.

Binding of HDAC7 to HIF-1α might lead to a conformational change resulting in facilitated binding of p300 and an increase in transcriptional activity [15]. p300 mediated c-Jun downstream targeted gene expression through the formation of c-Jun/p300 complex [22]. In this study, we found that transfection of either HDAC7 or p300 siRNA suppressed ET-1-induced protein–protein interaction among HDAC7, p300, and c-Jun. Moreover, ET-1-induced recruitment of HDAC7/c-Jun/p300 transcriptional complex to CTGF promoter was attenuated by transfection of HDAC7 siRNA. These results suggest that the presence of HDAC7 is required for ET-1-induced HDAC7/c-Jun/p300 transcriptional complex formation and recruitment to CTGF promoter.

Airway remodeling is characterized by subepithelial fibrosis [1] and contributes to progressive loss of lung function in asthma [29]. Myofibroblasts were reported to play a critical role in the progress of subepithelial fibrosis of asthma [24]. α-SMA is a hallmark of myofibroblasts, and its production is significantly increased in lung fibrosis [23]. In this study, HDAC7 was elevated in the lung of OVA-treated mice and was colocalized with α-SMA-positive cells in the subepithelial layer of the airway wall. We also found that HDAC7 participated in ET-1-stimulated CTGF and α-SMA production in human lung fibroblasts. The results suggest that HDAC7 may mediate subepithelial fibrosis through the regulation of myofibroblast differentiation and may participate in the pathogenesis of OVA-induced airway fibrosis.

## Conclusions

**Fig. 8:**
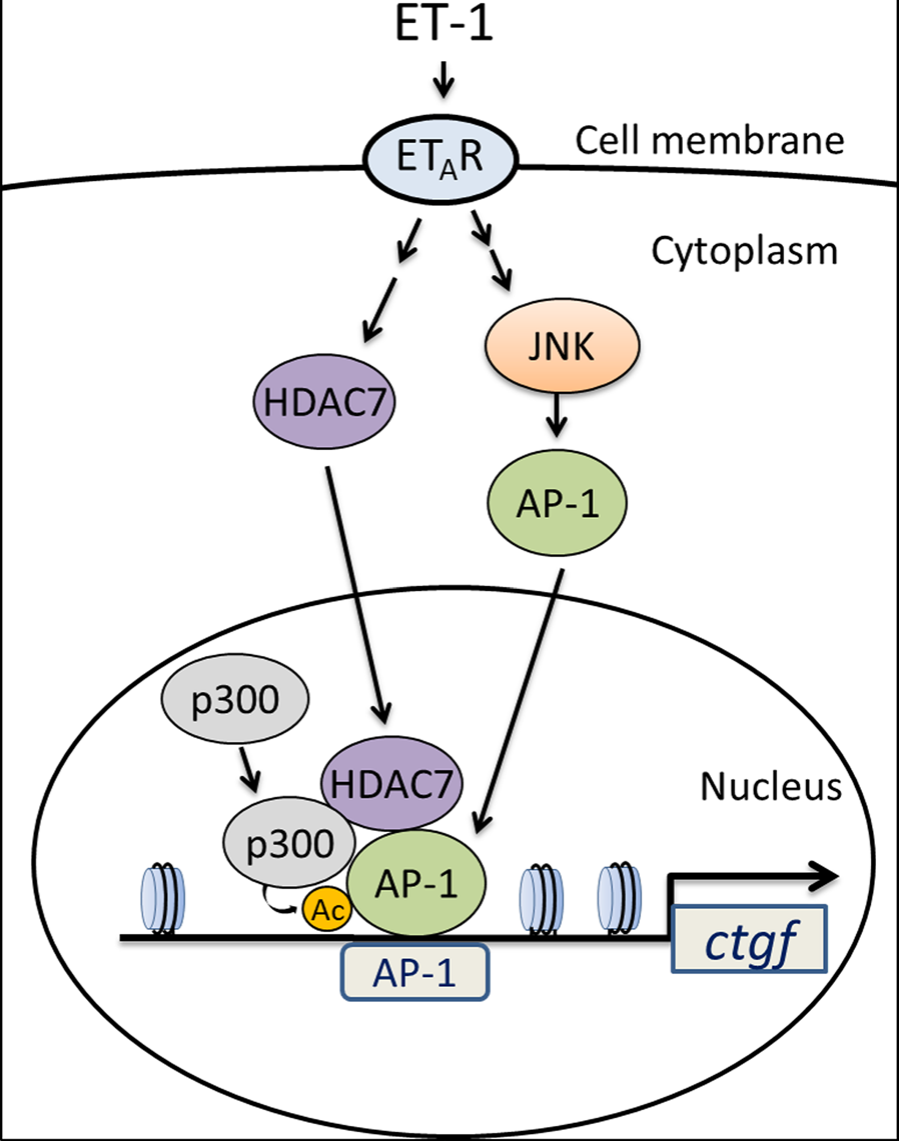
Schematic summary of how signal transduction by ET-1 induces CTGF expression. ET-1 acts on ET_A_R to activate the JNK signaling pathway, which in turn initiates AP-1 activation and ultimately induces CTGF expression in WI-38 cells. ET-1 stimulated the nuclear translocation of HDAC7 to form a transcriptional complex with p300 and AP-1, which in turn initiates c-Jun acetylation by p300. ET-1 thus recruits the transcriptional complex to CTGF promoter and eventually promotes CTGF production

In conclusion, the results of this study, together with those we previously reported [39], indicate that treatment of human lung fibroblasts with ET-1 causes activation of AP-1 and CTGF expression through two separate pathways: the ET_A_R/JNK/AP-1 pathway and the activation of HDAC7 and subsequent formation of the HDAC7/AP-1/p300 transcriptional complex. HDAC7 might act as a coactivator and mediate ET-1-stimulated CTGF production through the regulation of AP-1 activity. Under ET-1 stimulation, HDAC7 recruits c-Jun and p300 to assemble a transcriptional complex, which in turn enhances the transcriptional activity of AP-1 through p300-mediated c-Jun acetylation. HDAC7 is required for the recruitment of HDAC7/AP-1/p300 transcriptional complex to CTGF promoter and eventually promotes CTGF production upon ET-1 stimulation. Figure 8 presents a schematic of the signaling pathway of ET-1-stimulated CTGF production. We also revealed that HDAC7 might play a critical role in the pathogenesis of an OVA-induced airway fibrosis model. Therefore, HDAC7 has the potential to be developed as a novel therapeutic target to reduce airway fibrosis.

## Data Availability

The datasets used and/or analysed during the current study are available from the corresponding author on reasonable request.

## Abbreviations

AP-1: Activator protein-1
BSA: Bovine serum albumin
ChIP: Chromatin immunoprecipitation
COPD: Chronic obstructive pulmonary disease
CTGF: Connective tissue growth factor
ECL: Enhanced chemiluminescence
ECM: Extracellular matrix
EGF: Epidermal growth factor
ET: Endothelin
FBS: Fetal bovine serum
HAT: Histone acetyltransferase
HDAC: Histone deacetylase
HIF: Hypoxia-inducible factor
HRP: Horseradish peroxidase
IF: Immunofluorescence
IP: Immunoprecipitation
IPF: Idiopathic pulmonary fibrosis
JNK: C-Jun N-terminal kinases
MEM: Minimum essential medium
NEAAs: Non-essential amino acids
OVA: Ovalbumin
PBS: Phosphate-buffered saline
PCR: Polymerase chain reaction
PVDF: Polyvinylidene difluoride
SDS-PAGE: Sodium dodecylsulfate polyacrylamide gel electrophoresis
siRNA: Small interfering RNA
α-SMA: α-Smooth muscle actin
TGF-β: Transforming growth factor-β
VEGF: Vascular endothelial growth factor
